# Penetrating neck trauma and the rising rates of deliberate self-harm: a single trauma centre experience

**DOI:** 10.1308/rcsann.2025.0072

**Published:** 2025-10-23

**Authors:** M Qasem, ME Ryan, T Davies, AJ Kinshuck

**Affiliations:** Liverpool University Hospitals NHS Trust, UK

**Keywords:** Penetrating neck injury, Mental health, Deliberate self-harm, COVID-19

## Abstract

**Introduction:**

Self-inflicted harm is a complex behavioural phenomenon and a manifestation of a variety of underlying factors. Penetrating trauma is increasingly common, with rising knife crimes, gun violence and terrorism-related ballistics in the civilian arena. The study aims to review the changing patterns of penetrating neck injury (PNI) over a period of six years in our major trauma service (MTS).

**Methods:**

This was a retrospective review of all cases of adult patients presenting with PNI to Aintree University Hospital (AUH) between January 2017 and January 2023. Cases were identified through medical coding and review of electronic medical records.

**Results:**

A total of 71 cases of PNIs were identified from January 2017 to January 2023, marking a 13% increase in comparison with previous reports. Presentations due to deliberate self-harm (DSH) rose from 27% to 51%, representing an increase of 89% between the two time frames (*p*=0.0008), peaking at 77% of total presentations during the first wave of COVID-19. The prevalence of mental health disorders increased by 65% during the six years, reaching peak levels at 92% in the year 2020.

**Conclusions:**

This study highlights the rise of self-inflicted PNI in the context of increasing prevalence of psychiatric disorders, demonstrating the importance of reallocation of healthcare resources for health promotion, prevention and treatment in mental health services.

## Introduction

Penetrating neck injury (PNI) is a term used to describe trauma typically caused by a sharp implement that breaches the deeper structures of the neck.^1^ The neck is notoriously complex, housing important vascular, neurological and aerodigestive structures that are relatively unprotected and susceptible to injury.^2^ Although PNIs represent a small proportion of all penetrating trauma cases, such injuries have the potential to cause significant morbidity and mortality.^3^ Aetiologies include interpersonal violence, deliberate self-harm (DSH), accidents and terror-related violence.^4^ The intricate diagnostic and therapeutic challenges associated with PNI necessitate collaborative input from multiple specialties, including Emergency Medicine, Radiology, Oral and Maxillofacial Surgery, Otolaryngology, and Vascular Surgery teams.^5^

Aintree University Hospital (AUH), based in Liverpool, is the single receiving site for adult major trauma in Cheshire and Merseyside, serving a core population of around 630,000 people. The Major Trauma Service (MTS) is a regional collaborative service between AUH and The Walton Centre for Neurology and Neurosurgery, serving a population of approximately 2.3 million in the North West region and beyond.

Penetrating trauma is increasingly common, with rising knife crimes, gun violence and terrorism-related ballistics in the civilian arena. The incidence of police-reported knife crimes increased from 797 in Merseyside in 2017 to 1,307 by the end of 2023, marking a 64% increase over the seven-year period.^6^ In parallel with the growing incidence of knife-related violence over the past decade, the influence of the COVID-19 pandemic on both crime rates and healthcare access must also be considered.^7^ Nationwide lockdowns were first enforced in March 2020 and remained, with varying levels of severity, until February 2022. The oscillating levels of lockdown disrupted social interactions, limited access to medical and mental health services, and significantly hindered economic activity, all of which had a detrimental impact on the psychological eudaimonia of the population.^7^ The prevalence of depressive, anxiety and insomnia symptoms displayed a significant increase in the UK relative to prepandemic epidemiological data.^8^

The combination of recent escalating violence and the psychological sequelae following the pandemic not only poses significant threats to public safety, but also presents unique challenges to the NHS. Self-inflicted harm is a complex behavioural phenomenon and a manifestation of a variety of underlying factors.^9^ Studies have established a link between self-inflicted injury and violent crime in psychiatric and forensic populations, in which people prone to impulsive aggression and self-harm are vulnerable to emotional dysregulation and a low tolerance to adversity.^10–12^

## Objective

Analysing trends in PNI presentations and the characteristics of people affected by violent injuries is crucial for addressing the root causes behind these incidents. In this study, we aim to examine the changing patterns of PNIs at our MTS over a period of six years.

## Methods

### Study design

Caldicott guardian approval was sought before accessing patient records. The audit was registered with the Clinical Audit Management team of Liverpool University Foundation Trust. A retrospective review of all adult patients presenting with PNI to AUH was conducted between January 2017 and January 2023. Clinical coding in our trust is in accordance with the International Classification of Diseases 10th Edition.^13^ Cases were identified through medical coding and review of electronic medical records. Patients presented to a variety of specialties, including Oral and Maxillofacial Surgery, Otolaryngology, and General Surgery; all were included in the analysis.

### Data extraction

A structured proforma was drafted to extract data relevant to the aim of this study. Data pertaining to the following were extracted: date of presentation, date of birth, age at presentation, gender, aetiology of injury, mechanism of injury, admitting specialty and previous mental health history. Data extraction was conducted by a single author (MQ).

### Outcomes

The primary outcome of this study was an assessment of the changing patterns of PNIs at our Major Trauma Centre, and comparing it with previous published records authored by the same institution.^14^ Of interest are the evolving rates of DSH in comparison with violent assault, while reflecting on the prevalence of mental health disorders in the context of the COVID-19 pandemic.

### Statistical analysis

Rates of DSH were compared with those of violent assault, and considered against published evidence. Correlation between the rate of DSH and the prevalence of mental health disorders was sought. Nonparametric data were analysed using Fischer's exact test, with *p*<0.05 considered to be of statistical significance.

## Results

### Patient demographics and aetiology

**Figure 1:**
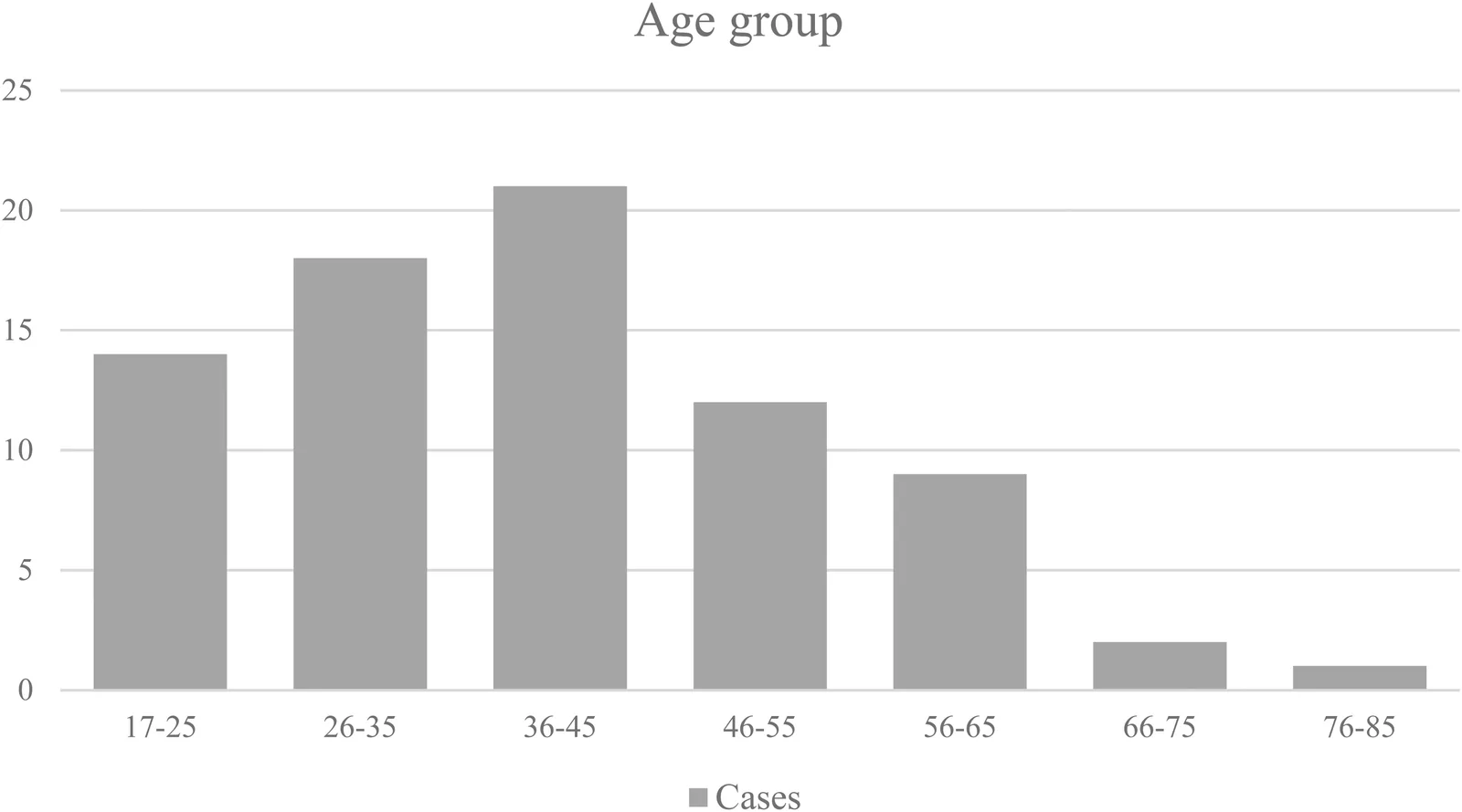
Age distribution of neck trauma patients. Incidence peak in patients aged 36 to 45 years.

Over the six-year period of analysis, 71 cases of PNI were managed at AUH. A male predominance was observed among the cohort, with 61 (86%) male and 10 (14%) female patients sustaining trauma to the neck. Average age at presentation was 40 years, ranging from 17 to 76 years (Figure 1). DSH was the disclosed aetiology in the slight majority of the cohort; 36 (51%) DSH versus 35 (49%) cases of alleged assault. No presentations were as a result of accidents or terror-related violence.

### Mechanism of injury

The mechanism of injury was most often a knife or a blade, accounting for 61 (86%) of all cases of PNI. Of the 36 cases of DSH, 83% were inflicted via a knife or blade, 14% via a razor and 3% using a screwdriver. A blade was the prevalent mechanism of injury in cases of alleged violence, responsible for 89% of said cases, whereas the remaining 11% were caused by broken glass. No injuries were sustained as a result of firearms.

### Trend of presentation

The total incidence of PNI was highest in 2017, with 21 cases of DSH and violent assault presenting in that year. Since 2020, the rate of PNI shows a steady decline, reaching its lowest at seven incidences per annum in the year ending December 2022. This trend is reflected in the incidence of PNI cases caused by alleged assault, being highest in 2017 at 15 per year and lowest in 2022 at 2 per year.

**Figure 2:**
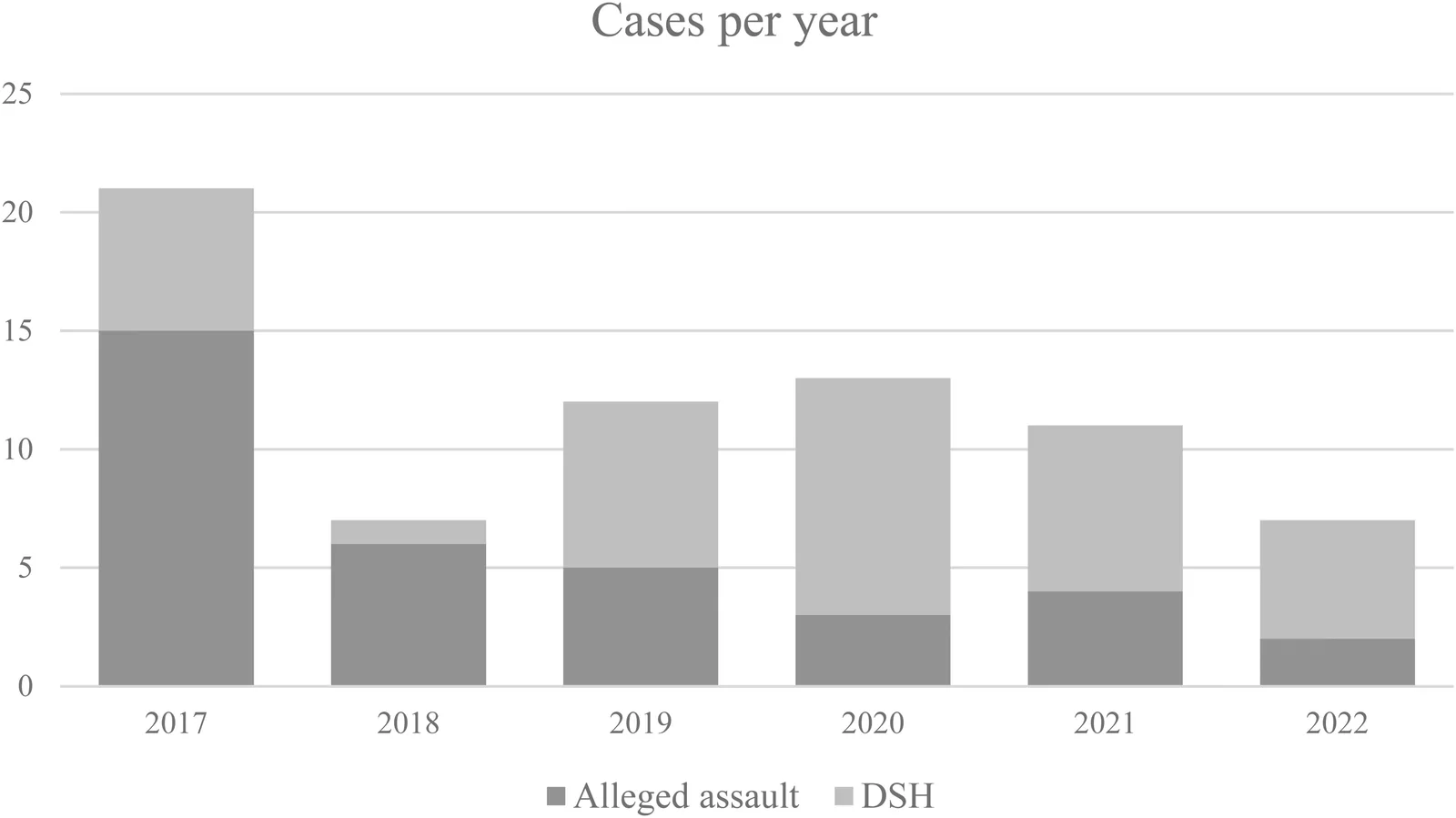
Trend of neck trauma cases managed per year

The proportion of PNIs caused by violent assault follows a general decline since 2017, with 15 cases being reported as caused by alleged assault in 2017 compared with only 2 in the year 2022. On the other hand, the contribution of DSH to the total case load of neck trauma displays a rising propensity, having accounted for 14% of PNI cases in 2018, and for 71% in 2022. The highest ratio of DSH to total PNI cases was during 2020, with 77% of presentations being self-inflicted (Figure 2).

### Imaging and findings

Of the 71 cases, 73% had a computed tomography (CT) or CT angiography (CTA) on presentation. Time to scan ranged from 14 to 177 minutes, with a mean of 24 minutes; 29% of imaging was performed at least partially due to associated injuries elsewhere, e.g. abdominal stab wounds, upper arm vascular injury, etc.

Exceedingly few images revealed significant findings of vessel injury; specific arterial bleed in two cases (internal carotid artery and vertebral artery), specific venous bleed in two cases (a facial vein injury and a ‘branch of internal jugular vein’ injury). One further scan reported an active extravasation from an unidentifiable source. Marked tissue emphysema indicative of a possible laryngotracheal injury was noted in five instances.

Of all the PNI cases over the six-year period, 46% underwent operative intervention of their injuries under general anaesthetic. Surgical exploration of the wound and washout was performed in 32 cases, and included repair of internal jugular vein, repair of internal carotid laceration, repair of facial vein, repair of marginal mandibular nerve, repair of recurrent laryngeal nerve, ligation of external jugular vein bilaterally and hemithyroidectomy. Interventional radiology intervention was performed in one instance that involved vertebral artery dissection. The rates of panendoscopy in the cohort was relatively small, being used in only 8% of PNI presentations, all of which suffered airway injuries.

Two patients had persistent lower motor neurological deficit at the time of their most recent follow-up despite operative repair; one with a unilateral recurrent laryngeal nerve palsy and another with marginal mandibular nerve weakness. No patients required a tracheostomy for airway protection, and instances of pharyngoesophageal injuries were managed conservatively following surgical exploration with favourable outcomes. Over the six-year period, one mortality (1.4%) was recorded as a consequence of PNI – a DSH patient injured by blade presented with hard signs and was taken to theatre without prior imaging. It was the intention of the authors to compare clinical outcomes with previously published data from our MTS; however, this was not reported previously.

### Psychiatric history

The prevalence of psychiatric disorders across our cohort was 66%; 77% of DSH and 55% of alleged assault presentations had formal diagnoses of mental health disorders before their injury. The variety of diagnoses in the cohort were as follows: anxiety or depression in 44% of patients, a variant of delusional disorders in 12% and a history of substance abuse in 9% of presentations. Of note, 15% of our cohort had previous documentation evidencing DSH preceding their index episode.

**Figure 3:**
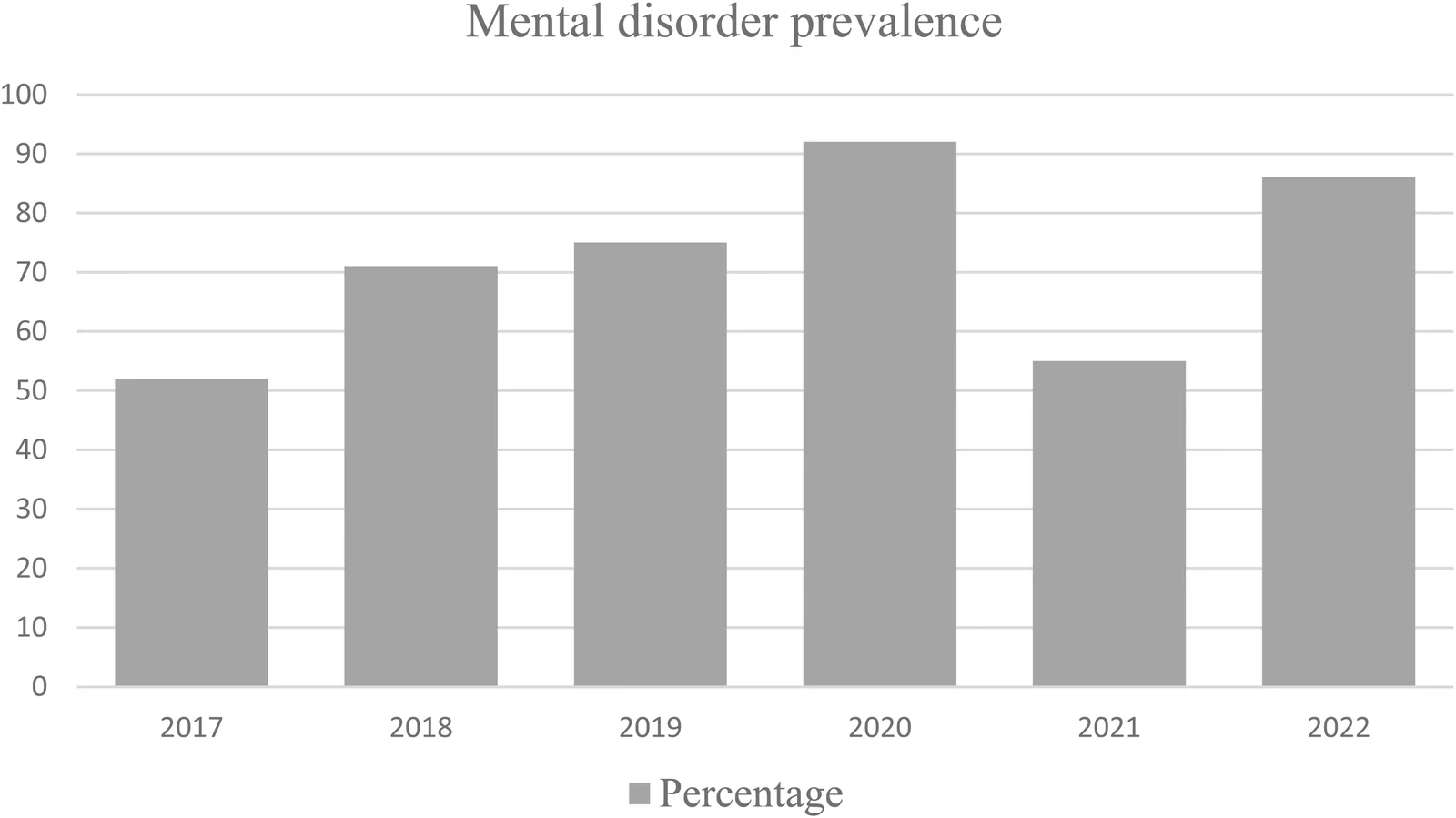
Percentage of the cohort with previous diagnosis of mental health illness.

The prevalence of mental health illness in the population presenting with PNIs displayed a steady increase in the years leading up to the first lockdown, peaking in the year 2020, with 92% of all PNI presentations having been diagnosed with a mental health disorder before injury. A second spike is noted in the year 2022, with said proportion reaching 86% of the total account of cases, in coincidence with the recission of COVID-19 restrictions (Figure 3).

### Comparison with previous years

A paper published by our institution in 2016 details the patterns of PNIs managed by our department between January 2007 and January 2013.^14^ Kasbekar *et al* describe 63 cases of PNI that presented over the six-year period.^14^ The median age at presentation was 33 years. The most prevalent mechanism of injury was reported to be a knife, accounting for 52% of the cases of PNIs across various aetiologies. Seventeen (27%) of the total account of injuries were the result of DSH. No effort had been made to collect data on the prevalence of mental health disorders.

**Figure 4:**
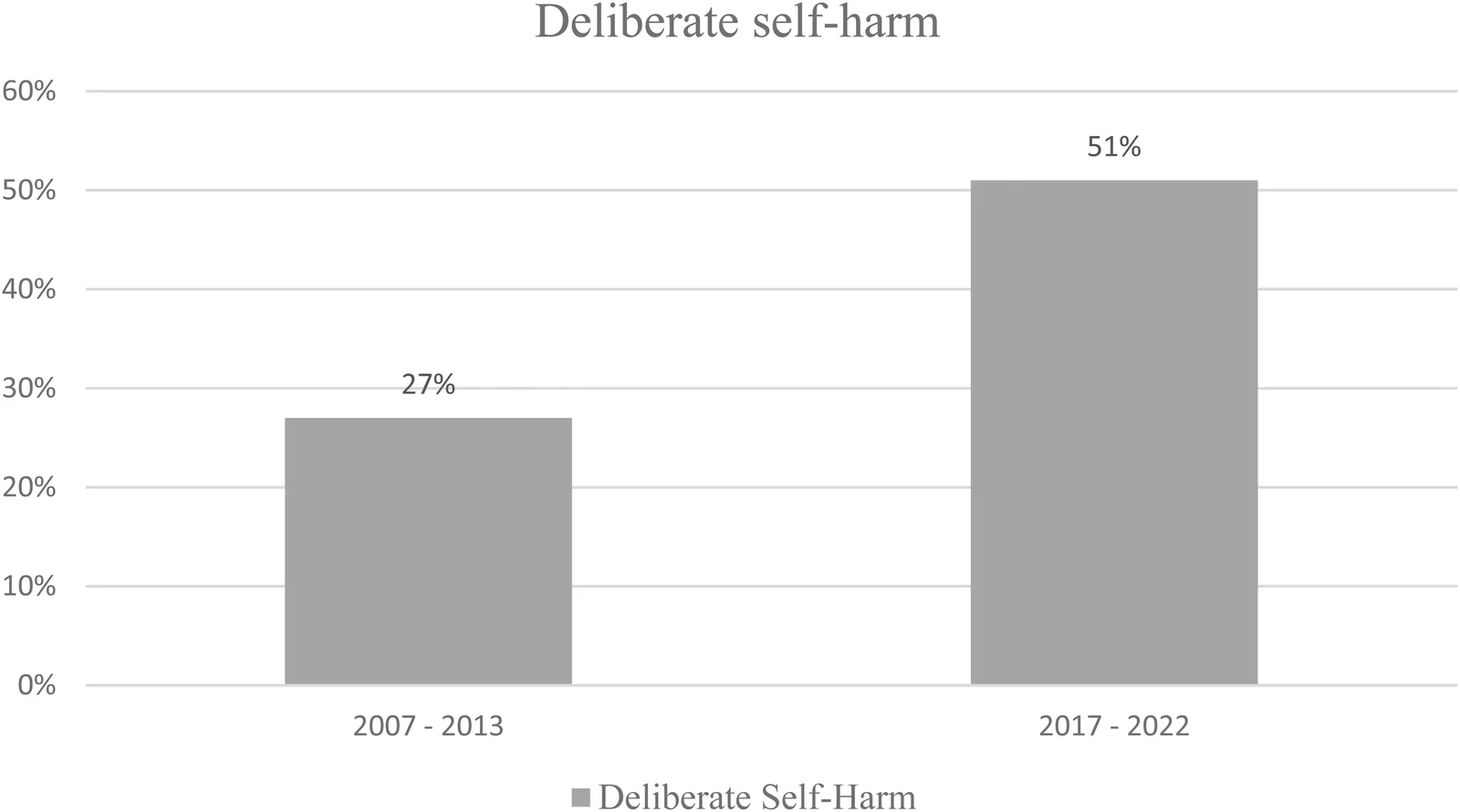
Proportion of PNIs as a result of DSH; 89% increase between the two cohorts (*p*=0.0008). DSH = deliberate self-harm; PNI = penetrating neck injury

In our cohort, 71 patients suffered a PNI, which represents a 13% increase over the findings of Kasbekar *et al*.^14^ Injury by knife or blade also demonstrated an increase of 65% since the previous audit, from 52% to 86%. Moreover, the proportion of those injuries sustained as a consequence of DSH portray a significant rise between the two time frames; rising from 27% to 51% (*p*=0.0008) (Figure 4).

## Discussion

The majority of penetrating neck trauma in the UK is of low velocity, often inflicted by a knife or sharp object.^14^ Although this mechanism of injury is a relatively uncommon reason for emergency presentation, the potential for morbidity and mortality is high, secondary to the vitality of the head and neck structures. We have demonstrated a peak in the incidence of penetrating neck trauma during the COVID-19 pandemic in 2020, and a subsequent downtrend thereafter. Furthermore, we identified a significant at rise in the rate of DSH as an aetiology for penetrating neck trauma when compared with a previous six-year review at our centre.^14^

DSH is often the result of complex interplay between biological, psychological and socio-economic factors.^15^ Mental health illness, especially depression, delusion disorder and substance use disorders are firmly established risk factors for DSH.^16,17^ A clear picture has developed of the mental health impacts of the waves of the COVID-19 pandemic in the UK at times where hospital admissions and mortality were more common, and lockdowns notably restrictive.^18^ The increase in symptoms of anxiety and depression were marked but often transient, peaking during periods of lockdown and returning afterwards to prepandemic levels.^19–21^ People who struggled with pre-existing mental health conditions, had lower educational attainment, lower income or resided in deprived areas were most affected and remained at higher risk throughout the restrictive policies and their aftermath.^22^ This is reflected by the increasing prevalence of mental health disorders in the cohort that sustained PNIs, irrespective of the aetiology of injury.

Interpersonal violence has profound consequences for its victims, perpetrators, healthcare planning and society as a whole.^23^ By January 2023, knife crime offences in the UK displayed a 75% increase compared with the year ending December 2013.^24^ Offences involving a knife or sharp instrument rose by 95%, from 26,694 in 2013 to 51,982 before the initial COVID-19 lockdown was enforced, reaching record levels since 1946.^25^ Of those reported cases, 44% were the consequence of violent assault with injury, or intent to cause harm.^26^ Street violence remains the primary cause of knife crime compared with all other reasons.^27^

Although the multiple guidelines and protocols that have been established in optimising the management of PNI as it presents to medical attention may have effectively improved morbidity and mortality, managing the root cause of this problem remains paramount. Multidisciplinary efforts are called for to address the wider concepts of public safety and justice, aiming to reduce the incidence of violent assault and identify highly vulnerable people before the occurrence of an injury. The 1998 Crime and Disorder Act, which aims to prevent crime, calls on collaboration among the police, local government and the NHS on joint crime-reduction strategies that require data sharing to inform targeted response. Although occasionally practised on an individual basis, the full potential for sharing of a whole agency dataset and linking to other organisations’ available data is rarely explored.^28^ The literature states that up to 77% of violent injuries treated in a hospital setting are not recorded by the police.^23^ Guidance by the General Medical Council and the Department of Health advises that anonymised information pertaining to all wounds inflicted in a violent attack by firearm or sharp object be forwarded to overcome barriers to data collection.^29^ Interdepartmental initiatives built on collaboration need to be implemented to facilitate identification of vulnerable people, improve safeguarding of those at risk and allow for targeted allocation of resources.

### Limitations

We are aware that the findings of this study may not truly reflect the incidence of PNIs in the region, and consequently the proportions of causative aetiology. We are unable to account for PNIs that do not present to medical attention, or are managed in district general hospitals. Previous diagnoses of mental health illness were collected from clinical records of the injury encounter, ambulance crew documentation and access to general practitioners’ records. There were instances where access to primary care records was restricted, and the clinical encounter notes did not report on psychiatric history. The findings of this project were presented and discussed at the monthly audit and governance meeting held in our department. Discussion has encouraged the department to prospectively collect all relevant clinical details, in accordance with the Cardiff model.^30^ With accurate and comprehensive data collection, the true incidence of PNIs, self-inflicted injuries and their prevalence in our population may be even higher than currently declared.

## Conclusions

This current study highlights the increasing incidence of self-inflicted PNIs in the context of worsening levels of psychiatric eudaimonia in the North West population of the UK. Efforts need to address the root cause of this phenomenon, mainly the rising rates of interpersonal violence, and the allocation of resources for mental health promotion, prevention and treatment of mental disorders.

## Data availability

The data that support the findings of this study are available from the corresponding author, MQ, upon reasonable request.
